# 3D-printed temperature and shear stress-controlled rocker platform for enhanced biofilm incubation

**DOI:** 10.1038/s41598-025-04575-3

**Published:** 2025-06-04

**Authors:** Daniel P. G. Nilsson, Krister Wiklund, Dmitry Malyshev, Magnus Andersson

**Affiliations:** 1 Department of Physics, Umeå University, Linnaeus väg 24, 901 87 Umeå, Sweden; 2 Umeå Centre for Microbial Research (UCMR), Umeå University, 901 87 Umeå, Sweden

**Keywords:** 3D printing, Biofilm, Design-build-test, Open-source, Laboratory rockers, CFD simulation, Bio-Rocker, Engineering, Fluid dynamics, Biofilms

## Abstract

Growing biofilms of thermophilic (heat-loving) and psychrotrophic (cold-tolerant) bacteria pose several challenges due to specific environmental requirements. Thermophilic bacteria typically grow between 45 and 80 $$^{\circ }$$C, while psychrotrophic bacteria thrive between 0 and 15 $$^{\circ }$$C. Maintaining the precise temperature and fluid conditions required for biofilm growth can be technically challenging. To overcome these challenges, we designed the Bio-Rocker, a temperature- and shear stress-controlled rocker platform for biofilm incubation. The platform supports temperatures between − 9 and 99 $$^{\circ }$$C, while its digital controller can adjust the rocking speed from 1 to 99$$^{\circ }$$/s and set rocking angles up to ±19$$^{\circ }$$. This ability, together with data from analytical models and multi-physics simulations, provides control over the shear stress distribution at the growth surfaces, peaking at 2.4 N/m$$^2$$. Finally, we evaluated the system’s ability to grow bacteria at different temperatures, shear stress, and materials by looking at the coverage and thickness of the biofilm, as well as the total biomass. A step-by-step guide, 3D CAD files, and controller software is provided for easy replication of the Bio-Rocker, using mostly 3D-printed and off-the-shelf components. We conclude that the Bio-Rocker’s performance is comparable to high-end commercial systems like the *Enviro-Genie* (Scientific Industries) yet costs less than $350 dollars to produce.

## Introduction

Laboratory shakers and rockers are important tools in chemistry, biology, and physics, providing dynamic environments for various experimental and industrial processes. In physics, these rockers aid in studying fluid and particle behavior under different motion regimes, providing insight into fundamental physical properties and processes^1^. In chemistry and biology, these rockers are important for the gentle mixing of solutions and aeration of a homogeneous suspension of cells, which is critical for ensuring consistent reactions and reproducible results. For example, the controlled movements offered by shakers and rockers facilitate an even distribution of nutrients, as well as fluid shear forces that promote optimal growth of bacterial cells and bacterial communities called biofilms^2,3^. By mimicking natural fluid dynamics, laboratory rockers provide a valuable platform for studying biofilm development in conditions that closely resemble their natural environments, thereby enhancing the reproducibility and reliability of biofilm research.

Biofilms are naturally forming aggregates of bacterial cells and spores, embedded in a protective matrix^4^. There is significant research into biofilms, as biofilm-forming bacteria present problems and challenges in both industrial and health-related sectors^5^. Biofilms provide bacteria with not only structural support but also substantial protection from environmental threats (chemicals, antibiotics, etc.)^6,7^. Moreover, biofilms formed by spore-forming bacteria, such as *Bacillus* and *Clostridia* species, develop resilient spore bodies that, when embedded in biofilms, are exceptionally difficult to neutralize^8,9^. The biofilms are also difficult to eradicate and can lead to persistent product contamination. Biofilms formed by spores and bacteria that express adhesion pili are believed to be even more robust, as the pili help reinforce the biofilm structure^10–13^. Therefore, it is crucial to understand how biofilms form, withstand harsh cleaning procedures, and persist under adverse fluid conditions.

Utilizing reactors and rockers allows for replicating these conditions in a controlled laboratory setting. Currently, there are only a few standardized reactors for this type of research, such as the Calgary Biofilm Device, the Center for Disease Control Biofilm Reactor, the Drip Flow Biofilm Reactor, and the Rotating Disk Reactor. Each offers unique advantages and limitations, as summarized in Table S1. However, some of these lack control over the shear stress or have small sampling surfaces, and precise temperature control is limited^14^. These limitations can be challenging in studies when specific environmental factors are required, such as growing cells and biofilms of thermophilic/hyperthermophilic (heat-loving) and psychrotrophic (cold-tolerant) bacteria. Thermophilic bacteria typically grow between 45 and 80 $^{\circ }$C, whereas hyperthermophilic prefer >80 $^{\circ }$C^15^. On the contrary, psychrotrophic bacteria thrive between 0 and 15 $^{\circ }$C^16^.

Biofilms also form at these extreme temperatures, as for example in the dairy industry, where the conditions can vary from refrigeration temperatures 7 $^{\circ }$C^17^ to as high as 80 $^{\circ }$C^18^, indicating conventional incubators do not have sufficient operating temperature range. Furthermore, some bacteria can even grow in highly saline environments, where the freezing point of water can be depressed well below 0 $^{\circ }$C. Another important factor for biofilm growth is the shear stress along the growth surface, like the inside of pipes or storage tanks. Biofilms have shown to adapt to hydrodynamic growth conditions, changing in both structure, density and adhesion properties, as well as there metabolism^19–21^. Thus, it is important for equipment to have control over the shear forces the biofilm is exposed to during its growth and for protocols to cultivate and study biofilms effectively. However, no commercial rocker platforms offer well-defined shear-stress applications and temperatures ranging from below 0 $^{\circ }$C up to 100 $^{\circ }$C, and most systems lack the capability to control the velocity and magnitude of the rocking motion accurately.

In this work, we present a 3D-printed laboratory rocker that incorporates temperature- and shear stress-control, capable of operating at both low and high temperatures. To test our Bio-Rocker, we utilized strains of *Bacillus thuringiensis* and *Bacillus cereus*, including a psychrotrophic variant originating from the dairy industry. We demonstrate that the growth and homogeneity of biofilms are strongly influenced by fluid shear stress from the rocking (Seesaw) motion, which can be accurately controlled in our rocker platform. We also perform computational fluid dynamic (CFD) simulations to assess the shear stress distribution along the sample surface. With the development of the Bio-Rocker, it will be easier to grow bacteria and understand the temperature and fluid conditions that promote the formation and persistence of biofilms, thereby contributing to more effective cleaning strategies. We provide all necessary manufacturing drawings and component lists to realize the build of the Bio-Rocker in a standard laboratory workshop.

## Design and construction

**Fig. 1:**
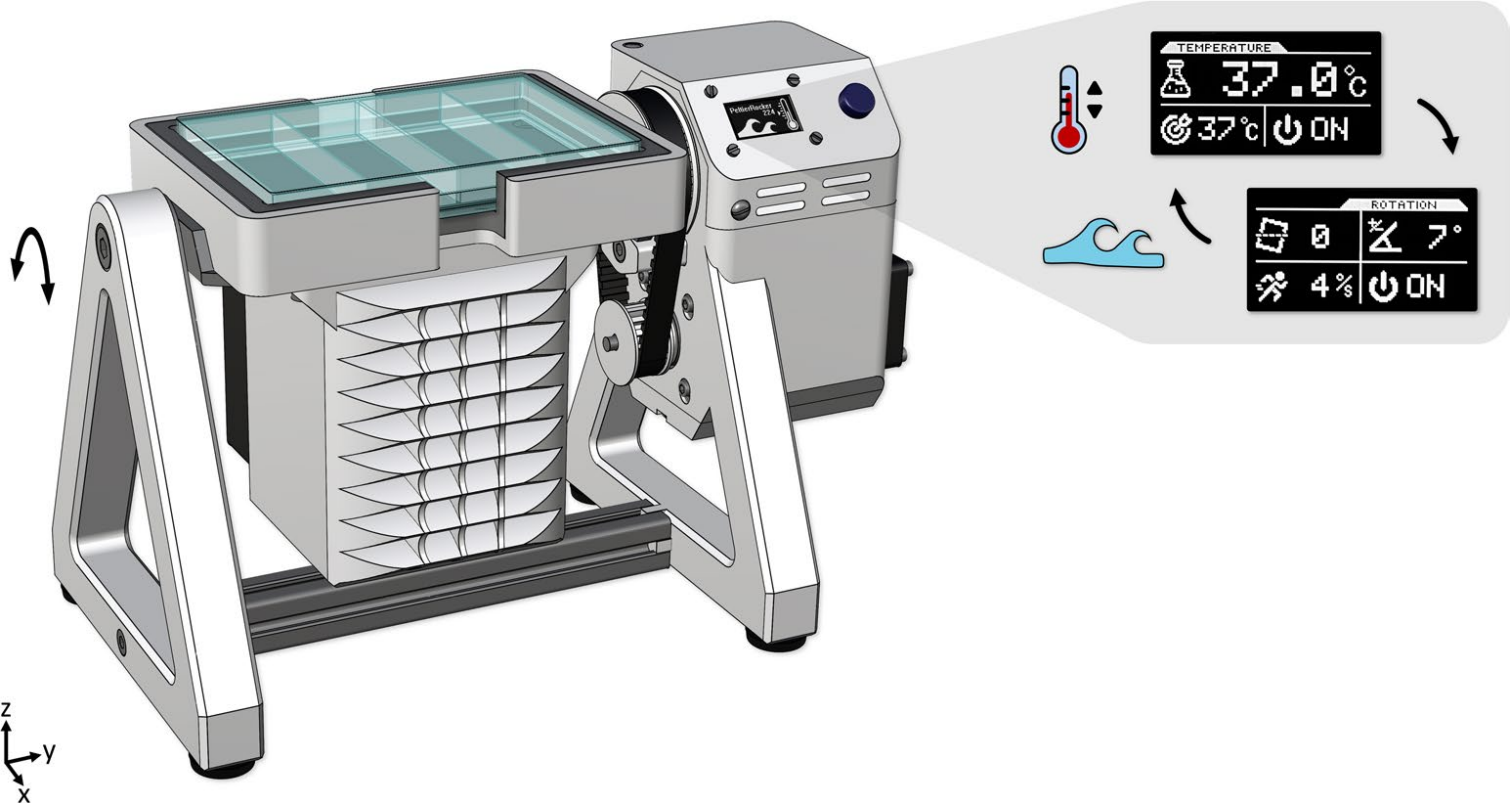
The design of the Bio-Rocker, whose major parts are 3D printable and feature digital motion and temperature controls. It is made to accommodate standard rectangular culture plates that are freely accessible from the top, while having a small overall footprint (150 × 275 cm). The user interface consists of separate pages for temperature and motion control that provide the users with interactive control and monitoring capabilities.

Many commercial systems fall short in their ability to accurately control the velocity and magnitude of the rocking motion, which is crucial for replicating real-world fluid conditions in biofilm research. To overcome this limitation, we developed a stepper-driven rocker mechanism that is controlled via a digital user interface. Additionally, while temperature control in these systems typically relies on cumbersome incubator enclosures, our approach simplifies this by directly integrating a thermoelectric (Peltier) module in the sample holder. This design ensures that the sample material maintains consistent contact with the temperature-regulated surface, providing an efficient temperature management method. The combination of precise mechanical motion and temperature control targets the specific needs of biofilm growth. The proposed design is shown in Fig. 1, together with its digital user interface and sample plate.

### Sample plate

We designed the sample holder to accommodate rectangular polystyrene culture plates with dimensions 127.8 × 85.5 × 14.5 mm (including the lid). For this study, we used a 4-well culture plate (*167063*, Thermo Scientific) that is made to accommodate 26 × 76 mm microscope glass slides, enabling the simultaneous growth of 4 separate cultures. This facilitates cell proliferation, viability, and adhesion assays on different substrate materials, such as standard-sized glass, plastic, or metal coupons. However, it is possible to also use culture plates with more wells, which would be advantageous for expanding the scale of the assays. For optimal performance, culture plates with flat bottom wells are recommended to enhance heat conduction, ensuring uniform temperature distribution essential for precise biofilm growth studies. Furthermore, these polystyrene plates have a surface treatment (Nunclon^TM^ Delta) that reduces the contact angle for water (ddH2O) from 90$^{\circ }$ to 70$^{\circ }$, and this may vary between manufacturers^22^.

The lids have a ventilation gap around the top perimeter of the culture plate, which allows condensation to move between wells during an experiment. However, for our study, we needed fully isolated replicates. Therefore, we developed a method to mold an airtight top membrane from polydimethylsiloxane (PDMS), a transparent and biocompatible silicone rubber. To do this, we first mixed 10 mL of elastomer base (*SYLGARD 184*, Dow Corning) with its curing agent at a weight ratio of 10:1. Then, the mixture was poured to an even layer into the culture plate lid (facing up) and the plate was placed into the lid (facing down). Finally, the PDMS was degassed using a vacuum desiccator before being cured in an oven for 2 h at 80 $^{\circ }$C^23^. After this process, the plastic lid can be removed, leaving a flexible and transparent membrane that a needle easily pierces when adding sample medium, or can be peeled off to gain access to the surface.

### Build guide

Building the Bio-Rocker requires off-the-shelf fasteners and electronics, as well as 3D-printed plastic parts and machined metal components. Detailed information on the parts and manufacturing drawings are provided in the supplementary materials, see Figs. S1–S7. All parts were designed using computer-aided design (CAD) software (*Rhino 6*, Robert McNeel & Associates) and the 3D models (.3dm-file), along with exported .stl- and .stp-files (AP 214, ISO 10303-21), are available in the resources^24^.

To ensure a smooth assembly process, it is recommended to first test the electronics on a solderless breadboard according to Fig. S2, before moving on to final assembly. Start by preparing a 50 × 70 mm two-sided prototyping board with 3 mm holes for mounting it on top of the MD13S driver board. Replace the connectors on the driver board with socket headers (2.54 mm pitch) and fit matching pin headers on the prototyping board. Place the Arduino microcontroller and the stepper driver (with heatsink) on the prototyping board with socket headers. Then, place the remaining components as space permits and connect them up using jumper cables (below or above the board), as shown in Fig. S3. Make sure that the Arduino USB port and the power supply connector are facing the opening in the control panel.

Next, assemble the fan shroud with its fan, heatsink, and thermistor, then mount it to the sample holder with the second thermistor and Peltier module using heat conductive paste. Assemble the right leg with its fan, rotation stop, stepper motor and drive wheel/belt, then press the ball bearings into their respective housings (interference fit) and feed the wires (Peltier, fan and thermistors) through the spindle. Mount the sample holder to the legs using the metal brackets and fasteners (the left bracket is mounted upside down), then connect the legs together using the aluminum strut (after threading the ends). Assemble the control panel with its screen and rotary encoder, mount the PCB stack in its housing and connect all the wires. Optionally, a spirit level can be mounted with adhesive to the sample holder, to aid at system startup.

### Programming guide

When the Bio-Rocker is fully assembled, it can be programmed and calibrated. The software, named *PeltierRocker*, was developed to run on resource limited platforms (like the popular *ATmega328P*) but still features a full graphical user interface (GUI). This was achieved by writing custom hardware routines for the temperature and motion control in C++ using an open-source editor (*Arduino IDE V.1.8.19*, arduino.cc). The IDE is also used to compile and upload the code to the microcontroller via the USB socket on the board. The software is available in the resources^25^ and requires some additional (open-source) libraries to be downloaded through the IDE, as described in the code. Before compiling and uploading, ensure the correct board type and port are selected. If compilation issues arise, pre-compiled code using the default parameters and pin configurations are also provided, and the uploading procedure is described in the supplementary materials.

When calibrating the Bio-Rocker, parameters need to be tuned for the motion and temperature controllers (see *config.h*). For the motion controller, the number of micro steps should match the settings on the DIP switch (16 used here) and the current limiter on the stepper driver should match that of the stepper motor (3/4 turn used here). The current limit can be tested by running the stepper at low speed and gradually increasing the current until the microsteps are consistent in size (either visually or audibly), but no more to avoid overheating. Further, the maximum rocker angle should match the 3D printed tilt stop (±19$^{\circ }$ used here), and the maximum acceleration settings can also be adjusted if necessary.

The Peltier module is controlled using pulse-width modulation (PWM) at the microcontrollers default frequency of 490 Hz (*Timer 2*), which is generally fast enough so that no thermal cycling is induced within the device^26^. If needed, tuning can be done of both the thermistors’ measurement circuit and the feedback control loop (PID) for the Peltier module. Before installing the temperature controller’s components, measure and confirm the manufacturer’s tabulated resistance values. Once fully assembled, a temperature sweep with a reference thermometer is advisable for fine-tuning the thermistor parameters. If needed, the thermal stability and response can be further refined by adjusting the PID parameters in the code, using the diagnostic view of the Bio-Rocker and following the guidelines from^27^.

### User guide

Before the very first use, press the rotary encoder button during power up (until the onboard LED starts to blink) in order to reset the user memory of the microcontroller (EEPROM). Short-pressing the button during operation toggles between setting menus (chosen menu is indicated with a blinking icon), while rotating the encoder knob changes the value. Long-pressing the button enters the diagnostic view for the temperature PID controller, see Table S2 for troubleshooting tips.

To set up for a run, level the rocker platform by hand before connecting the power supply, then prepare and load a culture plate in the sample holder. To use the temperature control (screen 1), set a desired target value (lower left) and turn the run state to *ON* (lower right). To initialize rocking (screen 2), begin by fine-adjusting the level control (upper left) until an even liquid film is achieved. Set the maximum rocking angle (upper right) and maximum speed (lower left), before turning the run state to *ON* (lower right). Alter the settings until the desired temperature and motion are achieved, using the reference data in Fig. 4 to find out the shear stress distribution.

## System performance and biofilm growth

When comparing our Bio-Rocker to commercial systems in Table S3, the *Enviro-Genie* (Scientific Industries) is the only rocker platform with comparable thermal capabilities. This commercial incubator-rocker offers a temperature range of 4 $$^{\circ }$$C to 75 $$^{\circ }$$C and a speed range of 1$$^{\circ }$$/s to 47$$^{\circ }$$/s, with a fixed rocking angle of ±10$$^{\circ }$$. This unit is priced at around $7000, weighs 36 kg and has a footprint of 3100 cm$$^2$$. In contrast, the Bio-Rocker has an extended temperature range of − 9 $$^{\circ }$$C to 99 $$^{\circ }$$C (or 35 $$^{\circ }$$C below ambient) and an increased speed range of 1$$^{\circ }$$/s to 99$$^{\circ }$$/s, with a variable rocking angle of ±1$$^{\circ }$$ to ±19$$^{\circ }$$. It weighs just 2.2 kg (excluding the power adapter) and has a significantly smaller footprint of only 440 cm$$^2$$. Although some of the other commercial systems comparable better to the Bio-Rocker in size, price and motion control, they all lack ability to cool a sample below ambient temperature, which is crucial when working with psychrotrophic strains. Below, we show how we evaluated its performance.

### Temperature evaluation

**Fig. 2:**
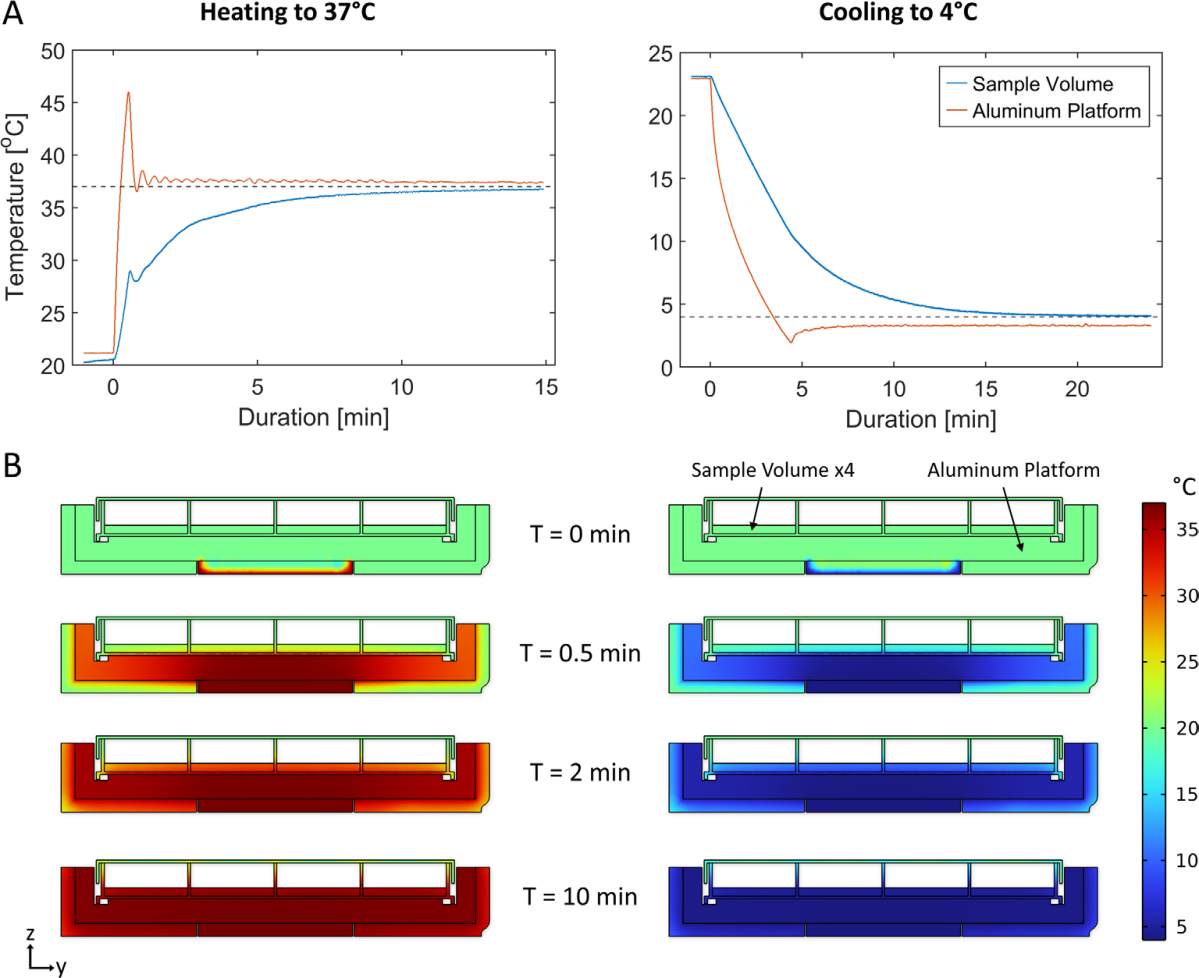
(**A**) Shows step response experiments on the Bio-Rocker while heating to 37 $^{\circ }$C (left) and cooling to 4 $^{\circ }$C (right), both starting from an ambient temperature of 21 $^{\circ }$C. The temperature was measured simultaneously inside the sample volume (7 mL water per well) and at the aluminum platform (sample holder). (**B**) Shows multiphysics simulations (front view) recreating the experiments in (**A**). The heat distribution at a central cut plane is presented at four different times while heating to 37 $^{\circ }$C (left) and cooling to 4 $^{\circ }$C (right) from 21 $^{\circ }$C.

The foremost contribution to biofilm formation is the temperature conditions in the growth medium. To evaluate our temperature controller’s performance, we attached external fast-response NTC sensors to the sample platform and recorded the temperature response. We filled each well with 7 mL of water before heating or cooling it from room temperature (21 $^{\circ }$C) to a fixed set-point, as seen in Fig. 2A. The NTC sensors were calibrated using a gallium fixed point cell (29.7646 $^{\circ }$C) to ensure an absolute accuracy of less than 0.1 $^{\circ }$C. The results indicate that the sample volume reaches a stable temperature of 37 $^{\circ }$C within 8 min or 4 $^{\circ }$C within 15 min, achieving a final temperature offset (steady state) of less than 0.2 $^{\circ }$C and 0.1 $^{\circ }$C, respectively.

Further, to evaluate the heat distribution inside the sample volume, we used finite element simulations (*COMSOL Multiphysics 6.1*, COMSOL AB) and modeled the heat flux for a stationary Bio-Rocker using the *Heat Transfer Module*. In our model, we represented the Peltier module as a constant temperature heat source/sink (no PID regulator), while incorporating natural convection throughout the rest of the model. This approach was appropriate as our primary interest was understanding the temperature distribution rather than the response time. The temperature distribution at different times can be seen in Fig. 2B on a cross-section plane through the middle of the four wells (each containing 7 mL of water). The simulation suggests that the sample volume should reach a uniform temperature in the equilibrium state, even though the Bio-Rocker and the water remained stationary. To verify this, we submerged a sensor in the outermost corner of the plate and heated/cooled the sample as before. After a similar period of time, we noted a discrepancy in water temperature of − 0.8 $^{\circ }$C and +0.3 $^{\circ }$C, respectively. However, this was measured for a stationary Bio-Rocker, and introducing fluid movement would likely reduce this discrepancy. So, lets evaluate how the rocking affects the motion of the fluid, and thus the growth of biofilm.

### Flow and shear stress evaluation

An important factor for the strength of the biofilm is the magnitude of the fluid shear stress acting on the growth surface. However, shear stress is challenging to measure on these small size scales. Therefore, both analytical and computational models have previously been used to calculate the shear stress in laboratory rockers for circular cultures plates^28^. The work by *T.B. Smith* et al. even resulted in an application for estimating shear stress using the inbuilt sensors of a smartphone (Android OS). However, these methods do not apply to the rectangular 4-well culture plate that we are using in the Bio-Rocker and the introduction of external motion tracking sensors is unnecessary thanks to our digital motion control.

**Fig. 3:**
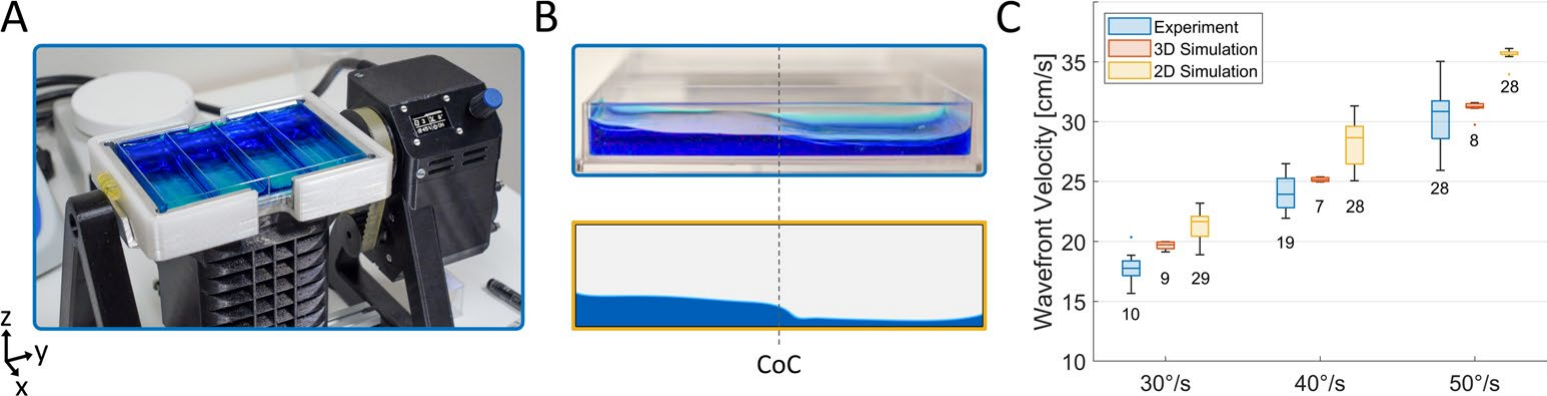
(**A**) Shows the Bio-Rocker with 9 mL colored water per well. When recording the wavefront, the sample holder was replaced with an open-sided holder. (**B**) Shows a side view (xz) of the culture plate when it is at full tilt (7$^{\circ }$) clockwise (top) and the corresponding snapshot from the 2D simulation (bottom), both in a co-rotating view frame. At this point, the wavefront passes the center of the channel (CoC) and moves toward the right. (**C**) Compares the wavefront velocity at CoC between experiments and simulations for different rocking speeds. The velocities were estimated by fitting position data from ± 2 cm around CoC and have a SE of less than ±0.2 cm/s. The boxes show the 0.25/0.75 quartiles and median value, the whiskers show the min/max value (excluding outliers), and the number of replicates is indicated below each box. Outliers are defined as being 1.5*IQR (interquartile range) away from the box boundaries.

To estimate the shear stress in the Bio-Rocker, we designed computational fluid dynamics simulations (CFD) in both 2D and 3D, as detailed in the supplementary materials. These were made to mimic the motion of the Bio-Rocker and the fluid flow within one of the culture plate wells, allowing us to visualize how the shear stress is distributed and how it changes for different rocking speed settings. Note that all subsequent work is done for a maximum rocking angle of ±7$^{\circ }$ from the horizontal, in accordance with previous research^1^. To validate the simulation’s flow behavior, we compared the velocity of the wavefronts produced by the rocking motion in the simulation to that of experimental measurements. These experiments were done using the Bio-Rocker with a culture plate well containing 9 mL of water and blue food dye (*2-60-033515*, Dr. Oetker Sverige AB), as shown in Fig. 3A. We recorded the water surface using a digital camera (*ILCE-6600* + *SEL1655G*, Sony Corporation) at 50 FPS and used this to calculate the velocity of the wavefront when it passes the center of the channel (CoC), as seen in Fig. 3B. The wavefront velocities were then compared for different rocking speeds in Fig. 3C, where the numbers below each box indicate the number of wavefronts recorded.

During these experiments, we noted that the culture plate showed such good wettability (low contact angle) that a thin liquid film was still present after the fluid had receded, seen as a bright region along the sample floor in Fig. 3A,B. At higher rocking speeds, wall friction further increased this behavior, allowing liquid to cling to corners and walls. This volume loss in the wavefront can explain why the experiment had a slightly lower velocity compared to the 3D simulation, where no liquid clinging occurred. The 2D simulation also seems to overestimate the velocity, which can be attributed to its lack of ability to simulate transverse waves that would help dissipate wavefront energy, thus increasing its velocity. However, to allow us to explore a broader range of sample volumes and rocking speeds within our computational load limits, we settled on using the faster 2D geometry for the bulk of our simulations. This gives us an upper limit of the shear stress distribution along the centerline of the channel. To verify this, we replicated two of our 2D simulations in 3D, as seen in Fig. S13 and Movies S1–S4. When comparing the total shear stress in Fig. S14, we see that the 2D simulations only overestimate the shear stress by 2.5% and 5.6% for rocking speeds of 4$^{\circ }$/s and 40$^{\circ }$/s, respectively. We also see that the 2D simulation accurately represents most of the channel width, and the effect from the side walls is only noticeable at the lowest rocking speeds.

**Fig. 4:**
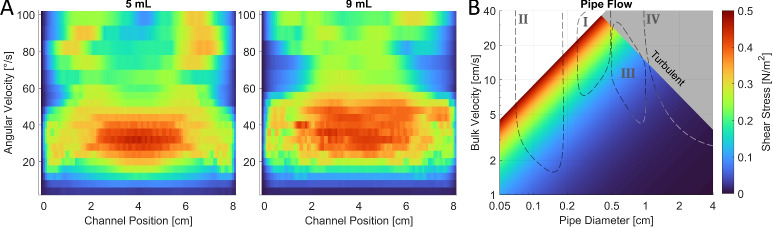
(**A**) Show wall shear stress data from 2D flow simulations of a culture plate well in the Bio-Rocker, filled with either 5 or 9 mL of water (37 $^{\circ }$C). Each row shows the shear stress along the centerline of the 8 cm long channel floor, as averaged over one rocking period (for a fully developed flow). The data provides quick translation between rocking speed settings and resulting shear stress when using the Bio-Rocker with a maximum rocking angle of 7$^{\circ }$. At this rocking angle, multiplying the angular velocity by $60s/(4\cdot 7^{\circ })=2,143$, gives the rocking speed in revolutions per minute (RPM). (**B**) Show theoretical wall shear stress for water (37 $^{\circ }$C) as a function of bulk flow velocity and pipe diameter. The color scale is the same for both (**A**) and (**B**) [N/m$^2$ = Pa = 10 dyne/cm$^2$], with the latter omitting values the Bio-Rocker cannot generate or where the flow becomes turbulent (Reynolds number > 2100). Regions I-IV show flow regimes for different scenarios where biofilm growth is problematic, and these include urethral and venous catheters, as well as hemodialysis and tap water pipes, respectively.

The complete shear stress data can be seen over many rocking periods and for speeds of 4–100$^{\circ }$/s in Figs. S10, S11. To summarize the findings, we averaged the shear stress at each position and over one rocking period for both 5 and 9 mL of water (a well holds 24 mL). By doing this time-averaging, each rocking speed is represented as a row in Fig. 4A, which shows how the shear stress is distributed along the channel length. Similar plots were done for the peak maximum values, as seen in Fig. S12. The highest average shear stress it can produce is 0.46 and 0.44 N/m$^2$, and the highest peak shear stress is 2.4 and 2.2 N/m$^2$ for 5 and 9 mL, respectively. Note that this occurs between 25 and 50$^{\circ }$/s, which is caused by a resonance between the wavefront and the rocking action that makes the wavefront more pronounced. We also noticed this during the wavefront experiments in Fig. 3. This can be verified by calculating the wavefront velocity required for resonance as $\nu = \omega L/2\alpha$, where $\omega$ is the rocking speed, *L* is the channel length and $\alpha$ is the maximum rocking angle. This yields velocities of 17, 23, 29 cm/s for rocking speeds of 30, 40, 50$^{\circ }$/s, respectively, similar to what we saw from the experiments.

To directly compare the shear stress conditions of the Bio-Rocker with that of a steady-state laminar flow in a circular pipe^29^, we used the empirical formula $\tau = 4 \; \upmu Q/\pi R^3$, where *R* is the pipe radius, $\mu$ is the dynamic viscosity of the fluid, $Q = \pi R^2 U_{mean}$ is the volumetric flow rate and $U_{mean}$ is the bulk flow velocity. Figure 4B shows the shear stress for water (37 $^{\circ }$C) at different flow velocities and pipe diameters, with the area where the flow transitions to the turbulent regime (Reynolds number > 2100) grayed out. This analysis allows users to configure the Bio-Rocker to generate shear stress that mimics real-world scenarios where biofilm growth is of interest.

Using urethral catheters as an example (region I), the flow velocities for 12 and 18 Fr sized catheter tubes starts at 8 and 15 cm/s for low bladder pressure (5 cm H$_2$O), reaching up to 35 and 60 cm/s at high bladder pressure (20 cm H$_2$O)^30^, respectively. Another use of catheters is in intravenous therapy to deliver 0.9% saline solution. These smaller venous catheters (region II) sized 16 and 24 G have flow velocities starting at 1.7 and 4.2 cm/s for low infusion-set heights (10 cm), but can exceed 130 and 200 cm/s when increasing the height to 100 cm^31^, respectively. Therefore, at low to moderate bladder pressures or infusion heights, these flows stay well within the laminar regime and the capability of the Bio-Rocker. In hemodialysis (region III), blood is circulated at low flow rates (200–400 mL/min) to minimize the risk of hemolysis^32^. It is transported through bloodlines of roughly 5 mm in diameter before reaching the 9 mm peristaltic pump tubing^33,34^, resulting in flow velocities of 17–35 cm/s and 5–11 cm/s, respectively. Another potential area of biofilm growth is in drinking water distribution systems (region IV). In the household, pipe diameters are commonly 1.0–2.5 cm and with maximum flow rates starting at 1 L/s^35,36^, corresponding to a flow velocity of 21–3.4 cm/s. All of which increases closer to the water processing plant. For industrial pipelines, the bulk velocity and pipe diameter are often large enough to generate turbulent flows. However, wastewater pipes often drain more slowly, especially when biomass is building up and causing partial blockages, so these could be of interest.

### Growing biofilms under varying shear stress and temperature

Biofilm formation and its characteristics are highly influenced by bacterial strain and growth conditions, with different strains having optimal growth temperatures. Also, the physical properties of biofilms are closely linked to the surrounding environment, including the amount of water and how it interfaces with air^37^. The water flow conditions also confer distinct physical traits to biofilms, compared to those developed under static conditions^38^.

To demonstrate our Bio-Rocker’s ability to grow biofilms, we cultivated biofilms from two different bacterial strains while varying the temperature and water flow (shear stress) over the growth surface. We utilized *Bacillus thuringiensis* (Bt407), a strain known for producing biofilms^39^, and *Bacillus cereus* (NVH 1534/24), a psychrotrophic strain isolated from refrigerated milk^40^. We start by taking 100 μL from a $10^7$ CFU/mL spore suspension as described in^41^. These $10^6$ spores were then added to each well together with 5 mL tryptic soy broth (TSB) (*Bacto*$^{TM}$, BD) and allowed to grow directly on the polystyrene culture plate, employing our silicone membrane method to seal the wells (as described previously).

The experiment encompassed three different flow conditions: static (0$^{\circ }$/s), slow (4$^{\circ }$/s) and fast (40$^{\circ }$/s), using the same maximum rocking angle (±7$^{\circ }$) as the simulations. Bt407 was grown at its optimal growth temperature of 30 $^{\circ }$C for 4 days, while the NVH 1534/24 was grown at 8 $^{\circ }$C for 10 days. Throughout the experiments, one of the four wells were left as a control containing only TSB. After incubation, we remove the liquid phase of the sample and use Gram staining to make the cells dark purple and easier to visualize^42^. This works on both Bt407 and NVH 1534/24, since they are both Gram-positive. We also performed a crystal violet assay on Bt407 replicates to quantify the total mass of the biofilm attached to the surface with optical density (OD) measurements and statistical analysis, as detailed in the supplementary materials.

**Fig. 5:**
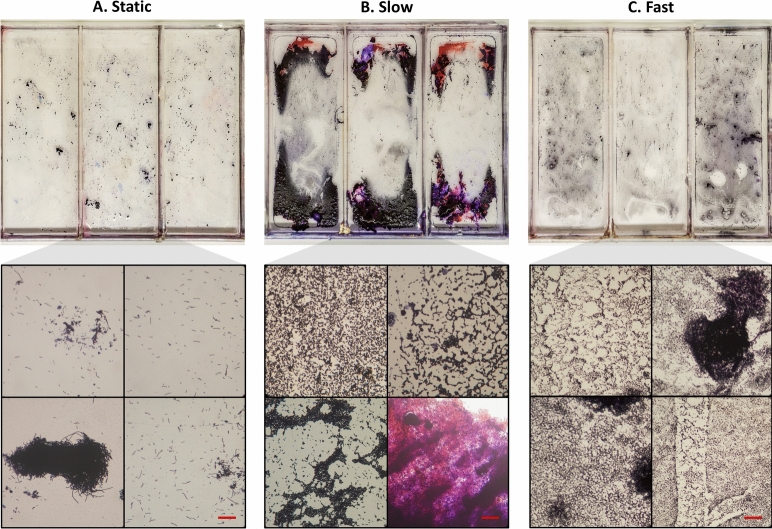
Gram-stained biofilms of *B. thuringiensis* (Bt407) grown directly on polystyrene culture plates and under three different flow conditions. (**A**–**C**) show three biological replicates of; static condition (0$^{\circ }$/s), slow flow (4$^{\circ }$/s) and fast flow (40$^{\circ }$/s), respectively. Underneath each culture plate are four microscope views of different parts of the sample, with a scale bar at 50 μm.

While Bt407 cells divide rapidly in TSB at 30 $^{\circ }$C, regardless of flow conditions, the resulting biofilms differ significantly. Under static conditions (0$^{\circ }$/s), most bacteria form pellicles, motile clumps not bound to any surface. Pellicles form at the air-water interface and are well known in *Bacillus* species^43^. However, they do not bind to any surfaces and are therefore lost during media removal, resulting in a biofilm OD of 0.114±0.026% (Fig. S15). Additionally, we note that the surface lacks a biofilm matrix, showing only scattered individual cells with rare cell clusters, as seen in Fig. 5A. In contrast, cells grown under slow flow conditions (4$^{\circ }$/s) show a completely different structure, with a much higher biofilm OD of 0.814 ± 0.036% (p = 0.0093). There is a thick optically opaque (dense) biofilm at the end of the wells in Fig. 5B, which covers 39 ± 2% of the surface, and no pellicles can be seen. Furthermore, the dense biofilm was relatively fragile and prone to detach during the staining procedure. Using our simulation results in Fig. 4A, we see that the dense biofilm mostly grew in regions with a shear stress lower than 0.03 N/m$^2$ (also seen from Figs. S13, 14). Closer to the middle, where the shear stress is higher, we instead see a monolayer of cells with varying density and a few larger cell clusters.

For the samples grown under fast flow conditions (40$^{\circ }$/s) in Fig. 5C, the biofilm OD is reduced to 0.248 ± 0.045%, significantly smaller than with slow flow (p = 0.0242). Here, the monolayer extends throughout the wells and the cells adhered so firmly to the surface as to be unaffected by washing. This monolayer is even unaffected by the peak shear stress of 2.4 N/m$^2$ at the channel ends, seen in Fig. S12, which corresponds to roughly 80 pN of force acting on each bacterial cell^44^. And these peaks remain high (> 1 N/m$^2$) for about 100 ms, much longer than the few ms it takes for bacterial pili to extend fully and no longer provide shock absorption^45,46^. Furthermore, there were no pellicle or other structures in this biofilm, and the liquid fraction of the biofilm appeared to be a homogeneous suspension. Notably, there was no biofilm along the corners of the wells for both slow and fast flows, as seen by a 2 mm border around the edges. This aligns with our previous observations from both experiments and simulations, suggesting that stagnant water accumulates and prevents biofilm formation there.

**Fig. 6:**
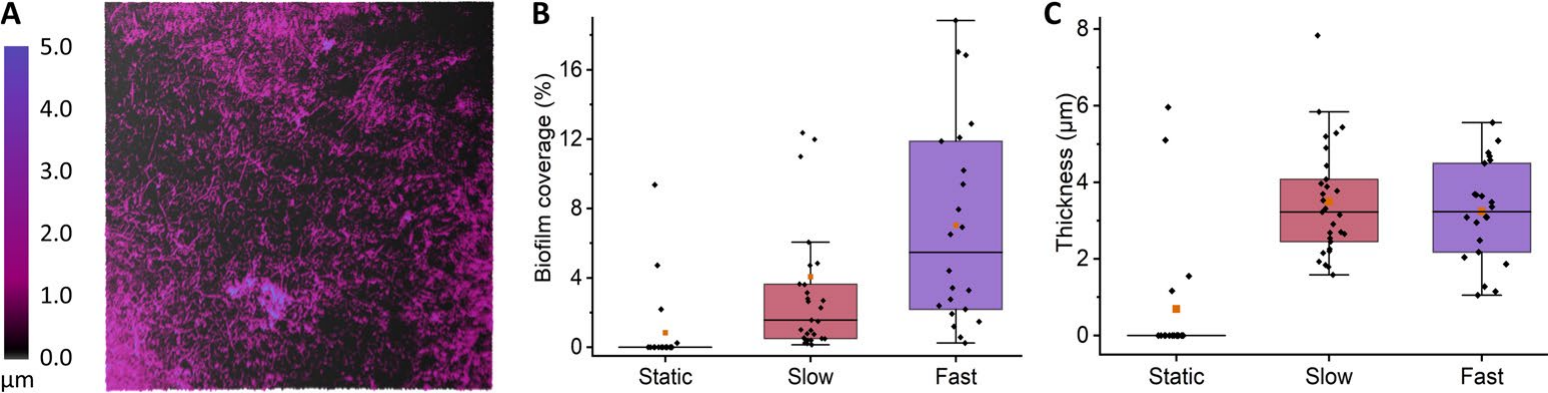
*B. thuringiensis* (Bt407) grown directly on polystyrene culture plates under the same conditions as the biofilms in Fig. 5. (**A**) Shows a representative height map (holography) of the monolayer biofilm present throughout most of the channels. (**B**,**C**) Show the coverage and thickness of the monolayer biofilm, based on $n_{static}$ = 20, $n_{slow}$= 29, and $n_{fast}$= 22 technical replicates.

To quantify biofilm thickness beyond the limitations of staining, we employed holographic imaging (*HoloMonitor*, Phase Holographic Imaging AB) on unstained replicates of Bt407 grown under identical conditions and seen in Fig. 6. This technique allowed us to assess biofilm coverage and thickness of up to 100 μm, which excluded some of the densest areas. Biofilms grown under static conditions revealed negligible biofilm coverage, with cells detected in only 4 out of 20 fields of view. Conversely, growing under flow conditions significantly increased coverage and the region at the channel ends showed thicknesses exceeding 100 μm. Interestingly, the average thickness of the monolayer biofilm remained comparable for both slow and fast flows, with values of 3.2±1.3 μm and 3.5±1.5 μm (p=0.82), respectively.

Next, we evaluated cell growth and biofilm formation with a cold-growing strain in the Bio-Rocker. Our experiments with the psychrotrophic NVH 1534/24 strain incubated at 8 $^{\circ }$C showed significantly slower growth due to the low incubation temperature. This is in line with other reports, which showed that at this temperature, bacterial division times are slowed considerably to around 12 h^47^. After 10 days of incubation, enough cells had grown to make the medium optically opaque, however, very few cells were attached to the surface. The resulting biofilm consisted of a sparse monolayer that was only slightly denser for the replicates grown under fast flow conditions, as shown in Fig. S8. Given the slow division rates, a longer incubation time may be necessary to form a dense biofilm at these low temperatures.

Our study demonstrates that the Bio-Rocker proved effective in cultivating biofilms under controlled conditions, revealing distinct structural differences in the biofilm produced on polystyrene culture plates. The Bt407 strain exhibited substantial biofilm formation under flow conditions, with notable differences in biofilm density and coverage. In contrast, the psychrotrophic NVH 1534/24 strain showed limited biofilm development at low temperatures, suggesting the need for extended incubation periods. These results are in line with previous studies, considering the higher shear force complexity of our system compared to continuous flow systems. As previously reported by Chang et al. and Liu et al., higher shear stress (>2.3 N/m$^2$) tends to produce smoother and denser biofilms, while low to moderate shear stress (>0.7 N/m$^2$) results in higher biomass, and static conditions resulted in very little adhered biofilm^19,20,48^. Meanwhile Wang et al., showed that at low shear stress, the smaller availability of nutrients limits the production of EPS and results in a loose and easily detached biofilm^49^, similar to what we observed under slow flow conditions.

### Growing biofilm on various materials

The ability to analyze biofilm formation on various materials is essential for assessing bacterial adhesion and biofilm development. To address this need, we developed an assay that simplifies the process of loading the Bio-Rocker with coupons made from various materials. This allows us to effectively assess how different surfaces influence bacterial adhesion and biofilm formation.

In these additional experiments, we utilized 26 × 76 mm coupons of glass (*631-0701*, ThermoFisher Scientific), stainless steel (AISI 304L/SS 2352), and polytetrafluoroethylene (PTFE) (*280-0575*, RS PRO), as well as PDMS (*SYLGARD 184*, Dow Corning) films. We repeated the previous *Bacillus thuringiensis* (Bt407) growth assay, but now at 37 $^{\circ }$C and only under slow flow conditions (4$^{\circ }$/s). To ensure that cell growth occurs on the top part of the sample, we used a thin layer of PDMS to fixate the coupons to the bottom of the culture plate. Each well was filled with 1 mL of PDMS before placing the coupons and sealing them with the top membrane (as described previously). After 4 days of incubation, the coupons were stained and removed for imaging. The results showed a consistent difference in biofilm attachment between the materials. Glass and stainless steel were the only materials that allowed a dense biofilm to form, as seen in Fig. S9. Similar to the uncoated polystyrene plate, this dense biofilm formed mainly at the end of the wells, while the rest of the sample had a sparse monolayer of cells. This monolayer was observed throughout all samples but at varying densities.

In conclusion, by developing an assay that simplifies the loading of the Bio-Rocker with various material coupons, we effectively assessed how different surfaces influence biofilm formation under shear stress conditions. Our results align with previous reports, demonstrating that polystyrene, glass and stainless steel are conducive to dense biofilm formation formed by many different species. In contrast, results on PTFE (Teflon) and PDMS showed that fewer biofilms were formed. These materials are indeed common in medical and industrial settings since their resistance to biofilm formation makes them suitable for applications where biofilm prevention is important. In a study by Zhu et al., they highlighted that the hydrophilicity of PDMS surfaces significantly affects bacterial adhesion and biofilm formation^50^. It was found that higher shear forces are required to inhibit bacterial adhesion on hydrophilic PDMS surfaces compared to hydrophobic ones. The study also demonstrated that at low flow rates, bacterial adhesion on PDMS surfaces is inhibited when the fluid flow exceeds a certain value indicating that hydrophilicity is the dominant factor affecting bacterial adhesion on PDMS surfaces. These findings further support the suitability of PDMS for applications where biofilm prevention is crucial.

## Conclusion

Since the introduction of laboratory rockers, they have played a crucial role in life science experiments. We have now enhanced its capabilities to better facilitate the study of biofilms, particularly those of thermophilic and psychrotrophic bacterial strains by increasing the range of possible temperatures and rocking speeds. In conjunction with our shear stress simulations, the Bio-Rocker’s digital temperature and motion control allows us to mimic real-world conditions with average and peak shear stresses up to 0.46 and 2.4 N/m$$^2$$, corresponding to 15 and 80 pN of force on single bacterial cells, respectively. Using a culture plate with four rectangular wells, we cultivated biofilms from two different bacterial strains under varying temperature and flow conditions, as well as on sample coupons of different materials. The resulting biofilms ranged from sparse monolayers to dense biofilms (> 100 μm), while using the same growth medium. Furthermore, to help promote a widespread adoption, the Bio-Rocker platform is made open-source and designed to use mostly 3D printed and off-the-shelf parts while costing less than $350 in materials and two days of active build time. As of writing, the platform has been used internationally and has accumulated over 10,000 h of combined run time. With this work, we hope to speed up the search for surface materials, treatments, or cleaning procedures that help combat biofilm growth where they are causing problems.

## Supplementary Information

Supplementary Information 1.

Supplementary Information 2.

Supplementary Information 3.

Supplementary Information 4.

Supplementary Information 5.

## Data Availability

All data are provided in the manuscript or supplementary information files.
