# Supplementary material for: 3D-printed temperature and shear stress-controlled rocker platform for enhanced biofilm incubation
Source: Sci Rep. 2025 Jun 4;15:19575. doi: 10.1038/s41598-025-04575-3 (PMC12137683; doi:10.1038/s41598-025-04575-3)
Supplement: Supplementary file 5 — Supplementary Information 5. [file 41598_2025_4575_MOESM5_ESM.pdf]

Development of a Temperature and Shear Stress-Controlled Rocker Platform for Biofilm Incubation Utilizing 3D Printing

Daniel P.G. Nilsson[1], Krister Wiklund[1], Dmitry Malyshev[1], Magnus Andersson[1,2]*

[1]Department of Physics, Umeå University, Linnaeus väg 24, Umeå, 901 87, Sweden.

[2]Umeå Centre for Microbial Research (UCMR), Umeå University, Umeå, Sweden.

*Corresponding author: Magnus Andersson (magnus.andersson@umu.se)

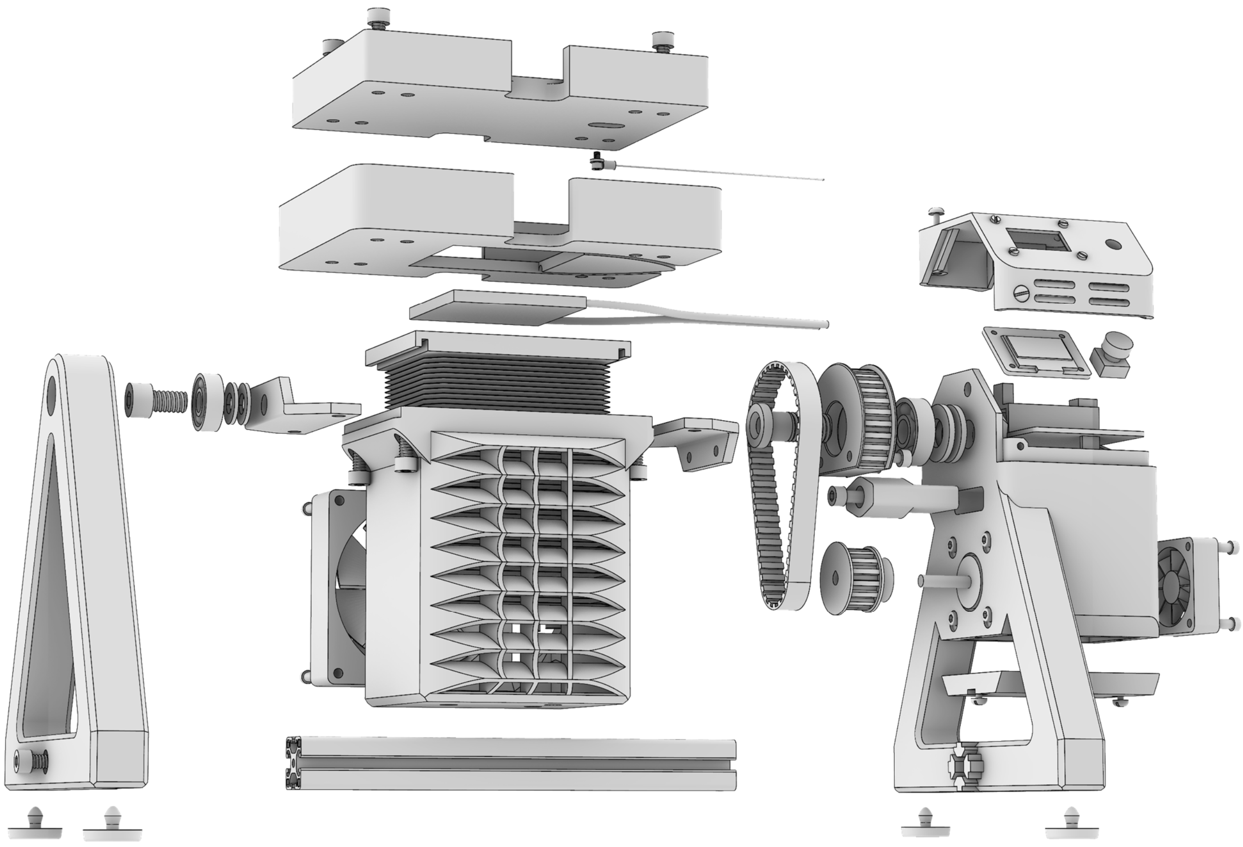

Fig. S1 An exploded view of the Bio-Rocker, showing all the major components and their relative positions. The CAD design file (Rhino3D V6), including exported STL manufacturing files is available in the resources.

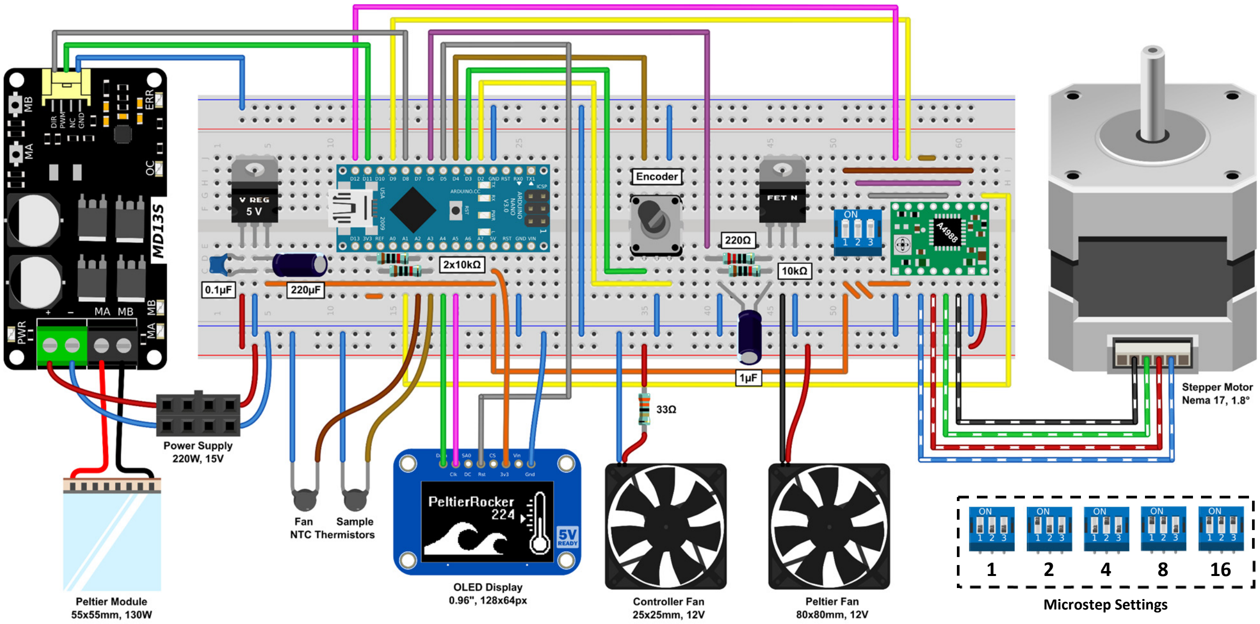

Fig. S2 The electronics and wiring needed for controlling the Bio-Rocker using the provided code. The setup uses an Arduino Nano microcontroller, an A4988 stepper driver and a Nema 17 stepper motor, an MD13S bi-directional driver and a 130 W Peltier module, an OLED display and a rotary encoder, as well as two cooling fans and two terminators. The DIP switch configurations for different number of microsteps are also shown (bottom right), more microsteps give higher stepper motor resolution. To fit in the controller housing, these components should be mounted to a prototyping board, either directly soldered or via JST connectors. To facilitate testing, the components are shown mounted to a solderless breadboard.

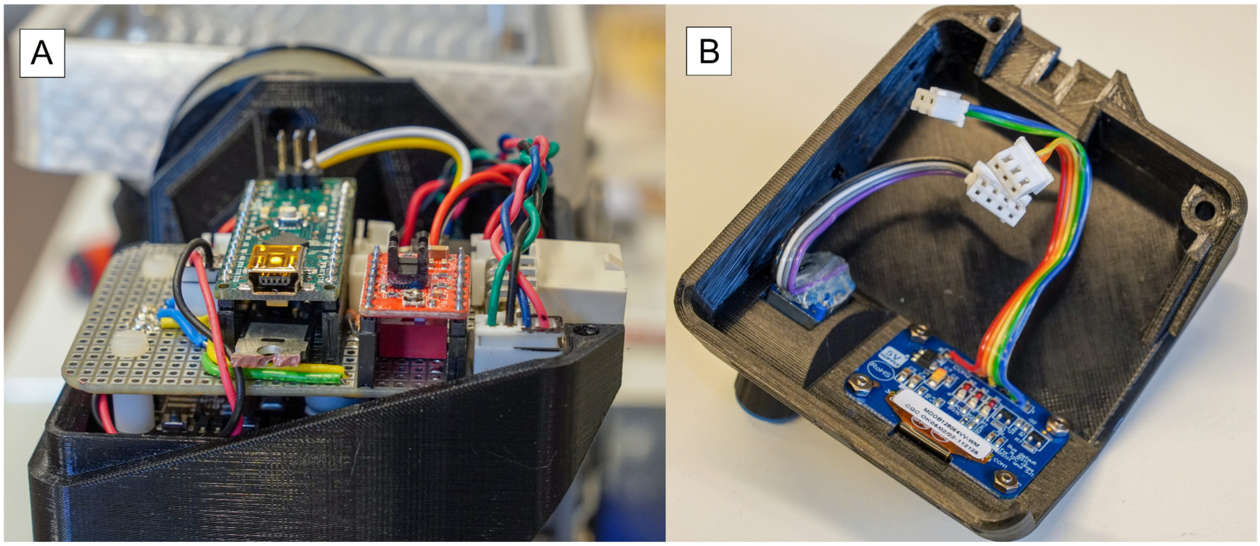

Fig. S3 The electronics and wiring fitted into the housing of the right leg. Panel A shows the prototyping board mounted on top of the motor driver board (PCB stack) and with all the components placed where space allows. Panel B shows the inside of the control panel, with its display and rotary encoder mounted.

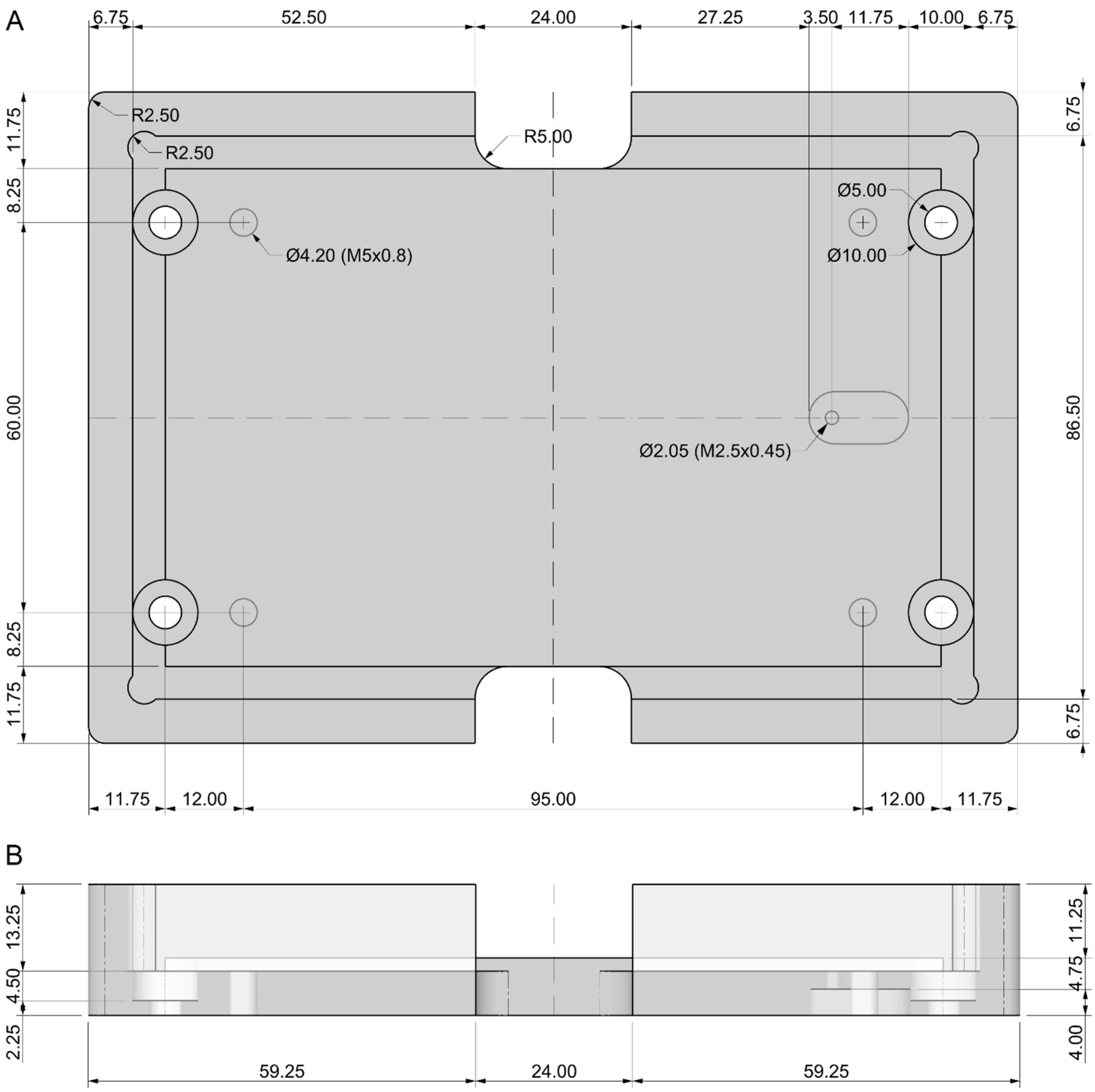

Fig. S4 Manufacturing drawing of the aluminum base that holds the exchangeable culture plates. The aluminum facilitates heat transfer between the sample and the thermoelectric module. The 4.2 mm blind holes should be tapped for M5x0.8 threads and the 2.05 mm blind hole for M2.5x0.45. For this part, start from a 20 x 100 x 150 mm solid aluminum bar and use a Ø5 mm end mill. Panel A and B shows the top and side view, respectively, with dimensions given in mm. The CAD design file (Rhino3D V6), including exported STEP manufacturing files is available in the resources.

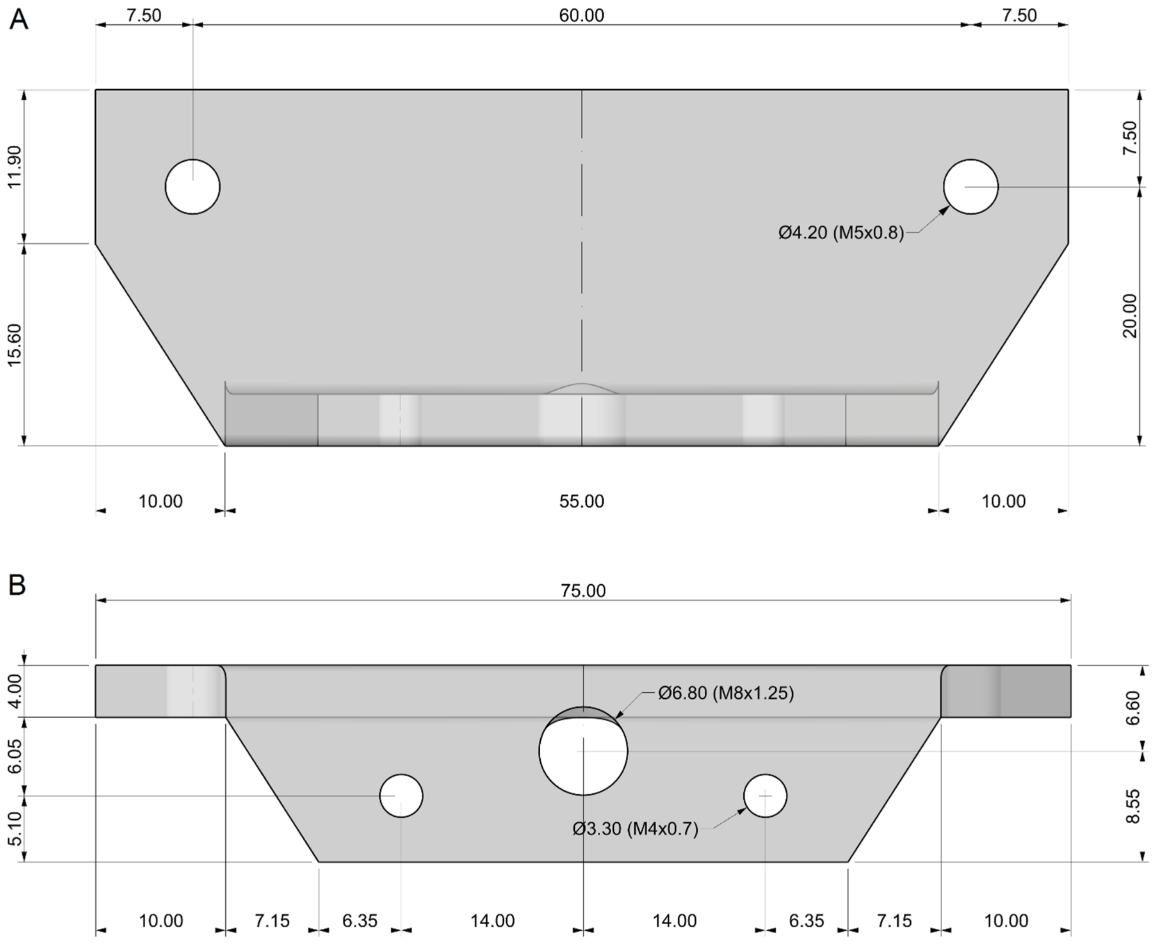

Fig. S5 Manufacturing drawing of the aluminum bracket that mounts the base plate to the rocker mechanism. Same design is used on both sides, with the left bracket mounted upside down. The 3.3 mm through holes should be tapped for M4x0.7 threads, the 4.2 mm through holes for M5x0.8 and the 6.8 mm through hole for M8x1.25. For this part, start from a 4 mm thick L-shaped aluminum profile. Panel A and B shows the top and side view, respectively, with dimensions given in mm.

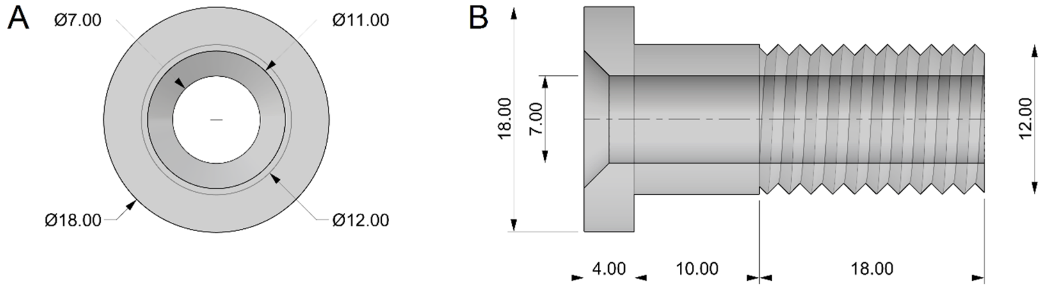

Fig. S6 Manufacturing drawing of the right-side spindle that connects the base plate to the rocker mechanism via a ball bearing in the UpperWheel.stl and allows for electrical wire feed-through. For this part, a M12x1.75 bolt can be drilled out and the head turned down. Using a socket head screw will allow tightening with a 10 mm hex wrench. Panel A and B shows the side and top view, respectively, with dimensions given in mm.

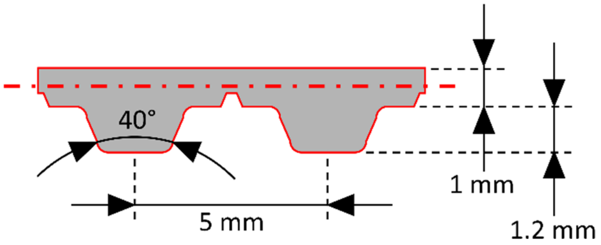

Fig. S7 Timing belt profile for SYNCHROFLEX® 10/T5/245SS (width/pitch/length), as used in the Bio-Rocker. Standard T-profile according to DIN 7721 with metric pitch and trapezoidal teeth.

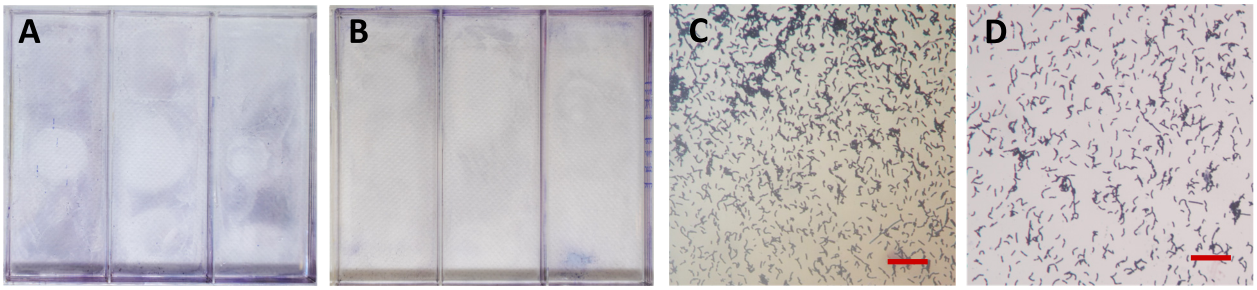

Fig. S8 Gram stained biofilms of psychrotrophic *Bacillus cereus* (NVH 1534/24) grown in 5 mL tryptic soy broth (TSB), directly on the culture plate and under different flow conditions. Panel A and B show three biological replicates for slow flow (4°/s) and fast flow (40°/s), respectively. Panel C and D show the monolayer biofilm at the center of the channel for slow and fast flow, respectively. Scale bar at 50 µm.

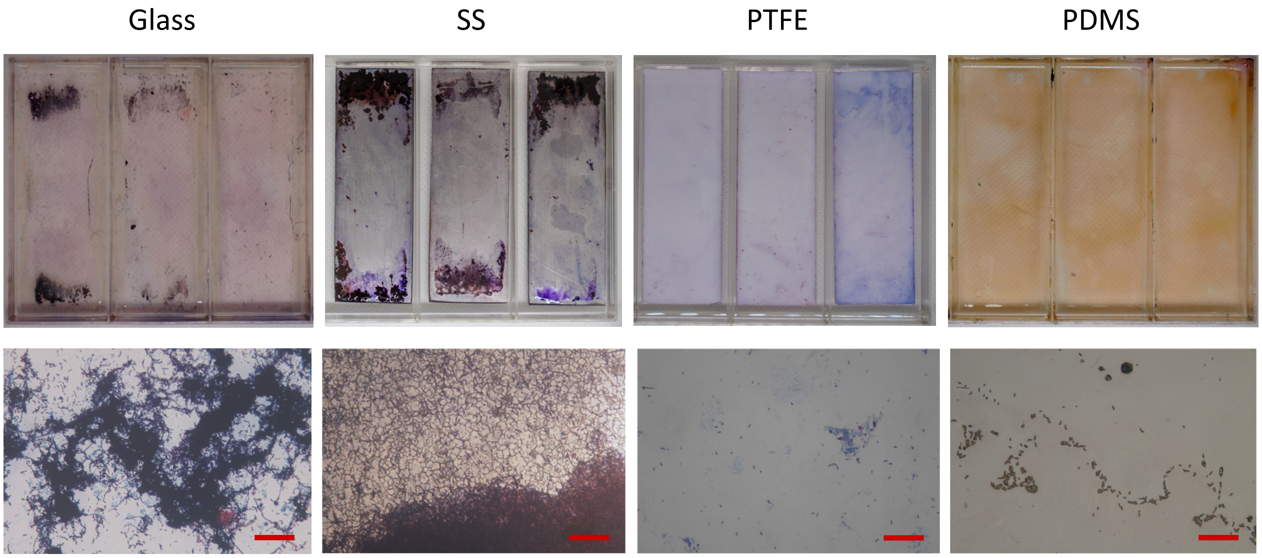

Fig. S9 Gram stained biofilms of *Bacillus thuringiensis* (Bt407) grown on sample coupons of different materials. Three biological replicates on glass, stainless steel (SS), polytetrafluoroethylene (PTFE) and polydimethylsiloxane (PDMS). Each replicate was grown for 4 days in 5 mL tryptic soy broth (TSB), at 37°C and with 4°/s rocking speed. Scale bar at 50 µm.

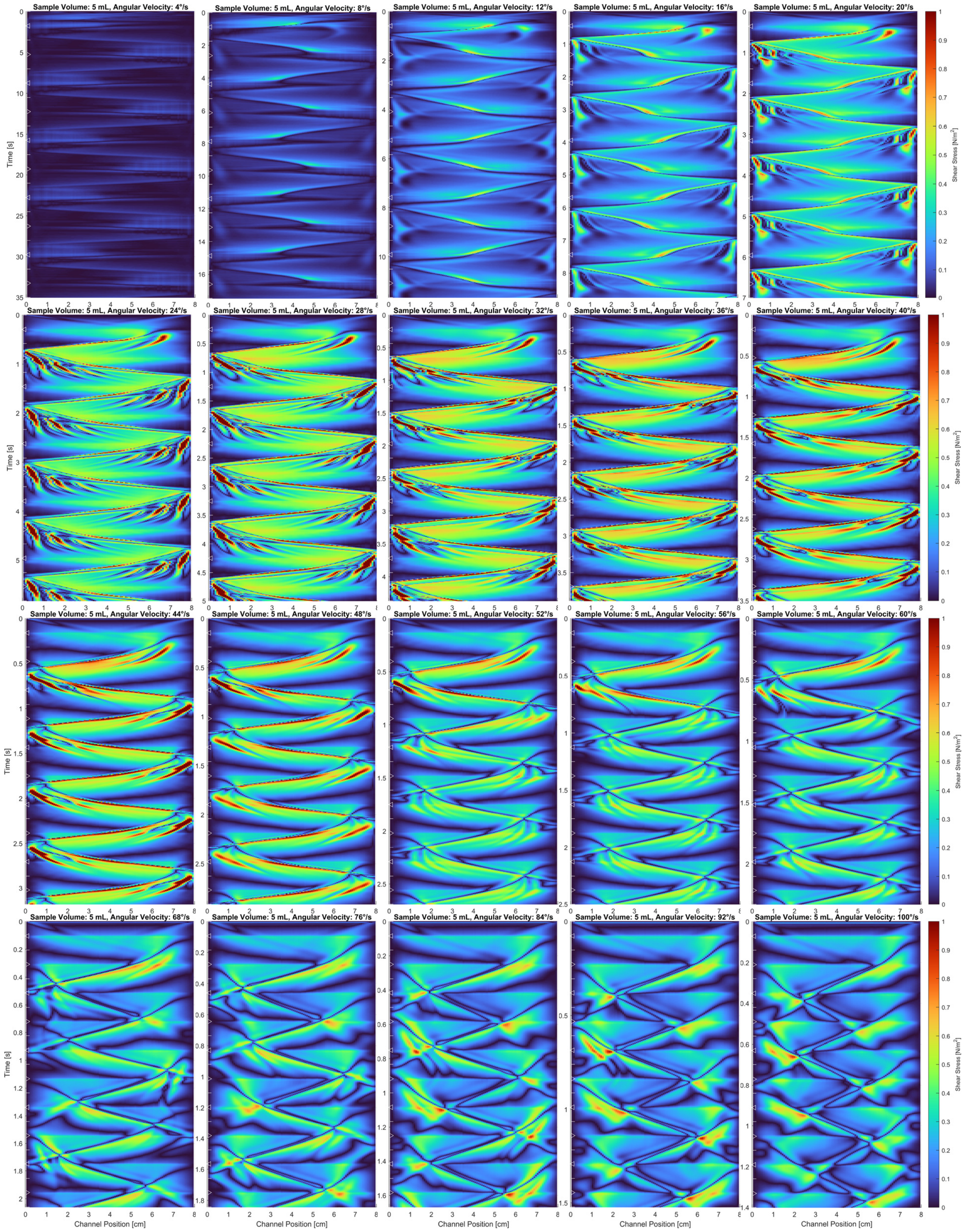

Fig. S10 Shear stress from 2D flow simulations of a Bio-Rocker culture plate well, filled with 5 mL of water (at 37°C). The vertical axis shows the evolution from a static to a fully developed flow behavior and the horizontal axis shows the distribution along the length of the 2D channel. Color scale is capped at 1 N/m^2.

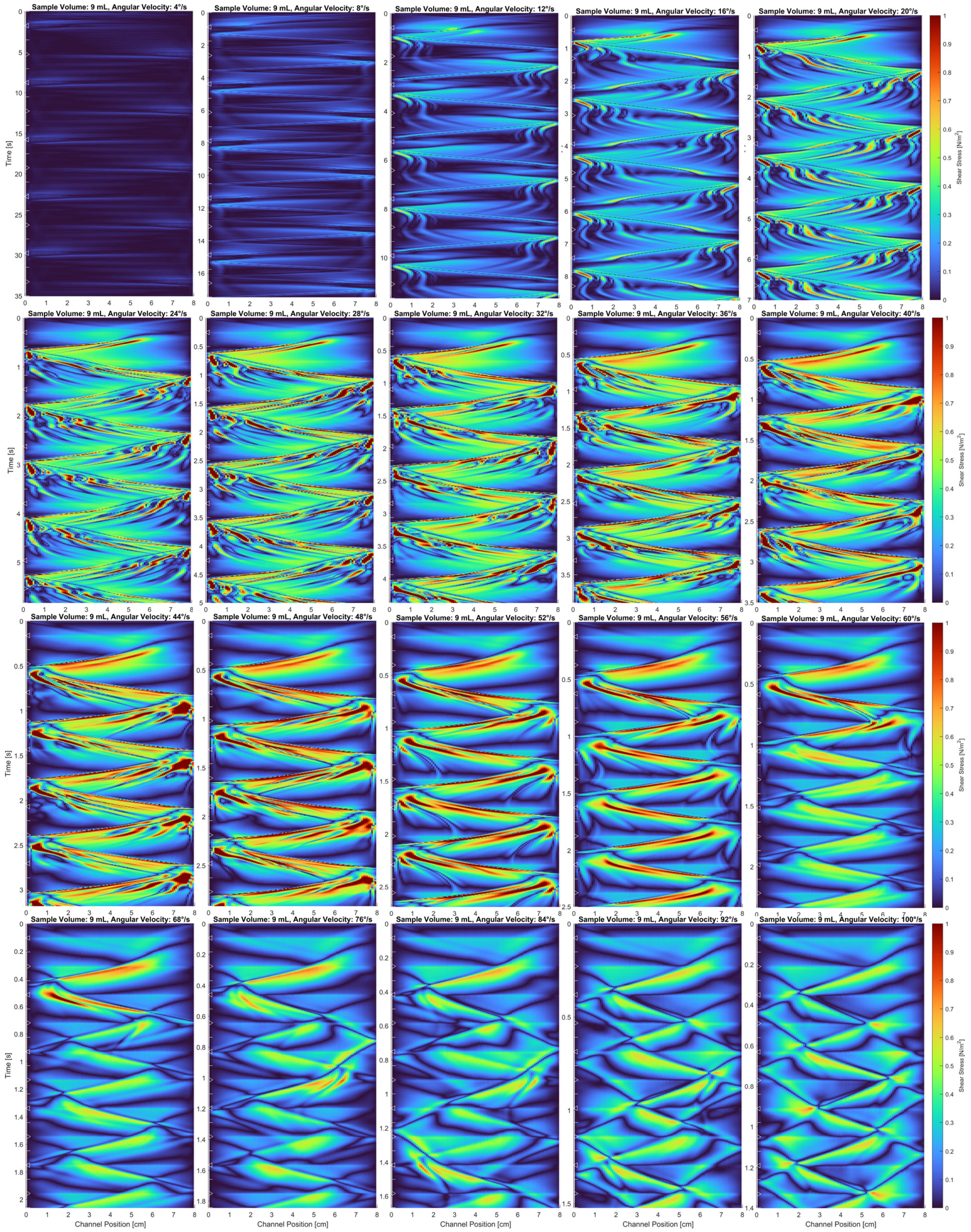

Fig. S11 Shear stress from 2D flow simulations of a Bio-Rocker culture plate well filled with 9 mL of water (at 37°C). The vertical axis shows the evolution from a static to a fully developed flow behavior and the horizontal axis shows the distribution along the length of the 2D channel. Color scale is capped at 1 N/m^2.

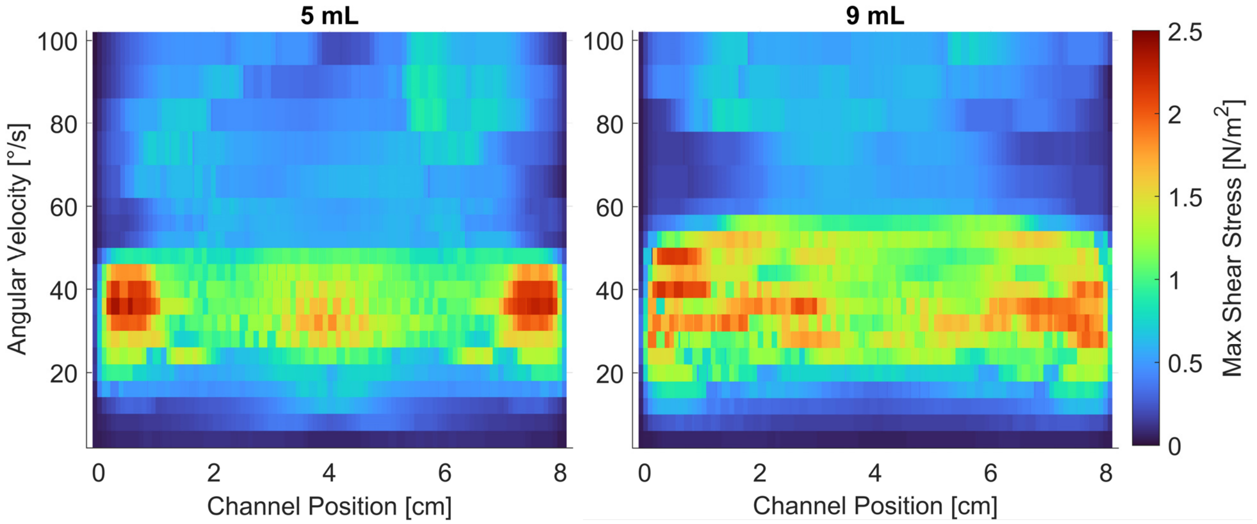

Fig. S12 Maximum instantaneous shear stress summarized from the simulation data in Fig. S10-12. Each row shows the wall shear stress along the centerline of the 8 cm long channel floor. The localized shear stress reaches its maximum of 2.36 & 2.18 N/m^2 at a rocking speed of 36 & 44°/s, for 5 & 9 mL respectively.

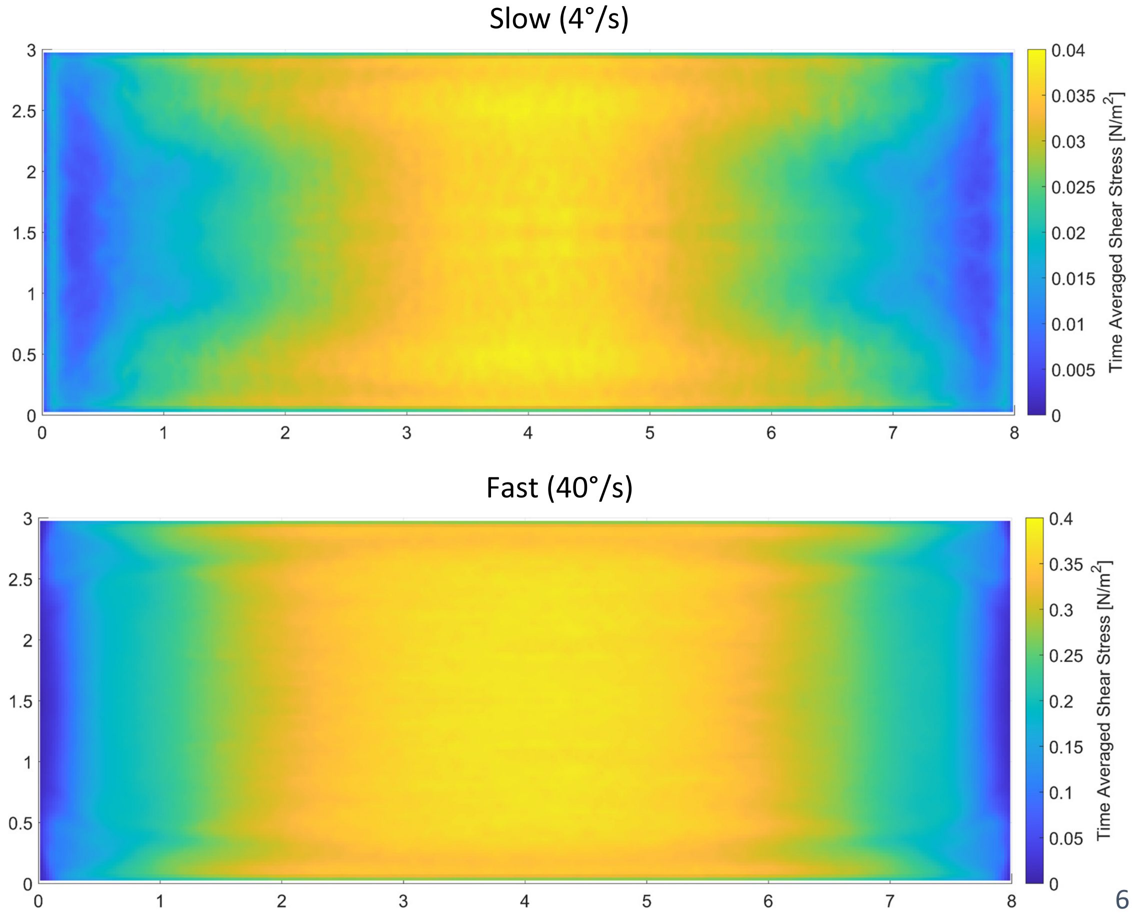

Fig. S13 Shear stress along the bottom surface of a well, averaged for one rocking period. This 3D simulation corresponds to the *Bt407* cells grown under slow/fast flow conditions and in 5 mL of water.

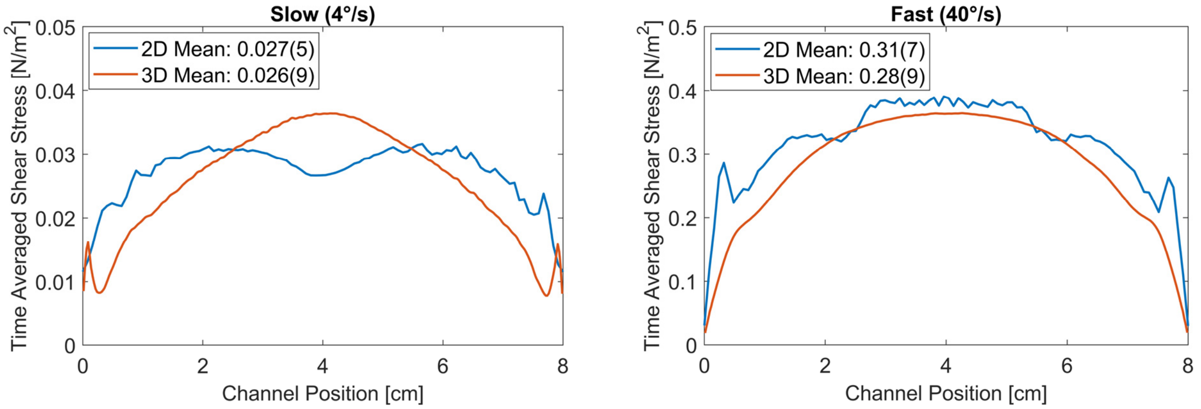

Fig. S14 Comparing the simulated shear stress distribution corresponding to *Bt407* cells grown under slow/fast flow conditions and in 5 mL of water, time averaged for a full rocking period. The 2D data is calculated from the first panel of Fig. S10, and the 3D data is an average along the width (y-axis) of the channel in Fig. S12 (top). The mean represents the average shear stress over time and the whole channel.

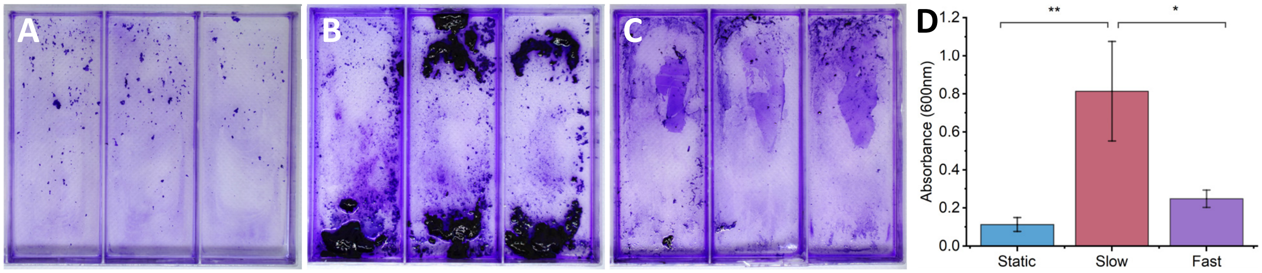

Fig. S15 Crystal violet-stained biofilms of *B. thuringiensis* (Bt407) grown directly on polystyrene culture plates and under three different flow conditions. Panel A-C show three biological replicates of; static condition (0°/s), slow flow (4°/s) and fast flow (40°/s), respectively. Panel D show optical density measurements, indicative of the biomass in each plate.

Table. S1 A summary of various biofilm reactors, their features and limitations.

Device	Features	Limitations	Ref
**Drip Flow Biofilm Reactor**	• Can continuously maintain nutrient concentration	• Only low shear<br>• No temperature control	[1]
**Calgary Biofilm Device**	• Uses breakable pins in standard 96 well plates<br>• Can adjust material for adhesion experiments	• No temperature or shear control	[2]
**CDC Biofilm Reactor**	• Rotating stirrer to for shear stress	• Size and geometry limits on coupons	[3]
**Rotating Disk Reactor**	• Sample coupons on a rotating disc<br>• Can continuously feed more nutrient broth	• Constant shear<br>• Size limits on coupons	[4]

Table. S2 Troubleshooting tips for common issues while building and using the Bio-Rocker platform.

Issue	Diagnosis	Solution
Uploading code	Test with example sketch *Blink.ino*	Install libraries and update board settings
Temp errors	- Check for erratic values	- Fix loose wire connection or replace sensor
	- If fan temp exceeds 85°C	- Clear obstructions and lower ambient temp
	- If sample temp exceeds 110°C	- Tune PID controller or swap Peltier polarity
Temp stability	- Fail to reach sample target temp	- Increase KP and decrease KI in *config.h* file
	- Unstable or fluctuating temp	- Decrease KP and increase KI in *config.h* file
Stepper motion	- Rocking velocity or angle incorrect	- Match DIP switch with MICRO_STEPS in file
	- Uneven or skipping steps	- Tune current limiter or replace tired motor
User interface	Display is dark or shows erratic pixels	Set SCREEN_ADDRESS according to datasheet
Contamination	If growth media spills from the plate	Clean platform with pure ethanol or IPA

Table. S3 A comprehensive list of commercially available thermal rockers and their specifications, sorted after unit price (ex VAT) as of May 2025. The *Signature* (VWR) is also sold as the *ISRK04HDG* (OHAUS), *02-217-761* (Fisherbrand), and *1000RS* (Talboys). *Price only include material costs

**Manufacture:**	**“DIY”**	**Ruicheng**	**VWR**	**Stuart**	**Scientific Industries**
**Model:**	*Bio-Rocker*	*TRS1000*	*Signature*	*SI30H*	*Enviro-Genie*
**Temp Range:**	-9 to 99°C	RT+5 to 65°C	RT+5 to 65°C	RT+8 to 80°C	4 to 75°C
**Temp Resolution:**	0.1°C	0.1°C	1°C	0.1°C	0.1°C
**Temp Accuracy:**	±0.2°C	±0.5°C	±0.5°C	±0.5°C	±0.2°C
**Rocking Control:**	Digital	Digital	Digital	Analog	Digital
**Rocking Speeds:**	0 to 46 rpm	0 to 60 rpm	1 to 50 rpm	5 to 70 rpm	1 to 35 rpm
**Rocking Angles:**	1 to 19°	0 to 15°	0 to 15°	4, 7 or 10°	10°
**Timer:**	No	1 min to 99 h	1 s to 160 h	No	up to 99 h
**Max Load:**	-	5 kg	4.5 kg	-	4.5 kg
**Weight:**	2.2 kg	12 kg	6 kg	24 kg	36 kg
**Footprint:**	440 cm^2	1 200 cm^2	1 200 cm^2	1 440 cm^2	3 100 cm^2
**Unit Price:**	$350*	$1 900	$3 400	$4 700	$7 000

Component List

The parts needed to build a Bio-Rocker are listed below and their assembly is shown in Fig. S1.

Off-the-shelf Parts:

- 1 pc - Nema 17 Stepper, 1.8°, 2.4 A (17H2A8423, MotionKing)
- 1 pc - Drive Belt, see Fig. S7 (10/T5/245SS, Contitech)
- 1 pc - PowerEdge CPU Heatsink (0D4730 or 0KJ582, Dell)
- 1 pc - Peltier Module, 55 x 55 mm (ET-127-20-15, Adaptive)

Supplementary Materials

- 1 pc - Heat Conductive Paste (HTCP20S, Electrolube)
- 1 pc - OLED Display, 0.96” (MDOB128064VV-WM, Midas)
- 1 pc - DC Adapter, 220 W, 15 V (VES220PS15, XP Power)
- 1 pc - Minifit Jr. Header, 2 x 4 pin, 4.2 mm (39-30-0080, Molex)
- 2 pcs - NTC Thermistor, M2 (NTCALUG03A103GC, Vishay)
- 2 pcs - ZH Connector Header, 2 pin, 1.5 mm (B2B-ZR, JST)
- 1 pc - A4988 Stepper Driver & Heatsink (1182, Pololu)
- 1 pc - Arduino Nano Microcontroller (A000005, Arduino)
- 1 pc - Bi-directional Driver Board, 13 A (MD13S, Cytron)
- 1 pc - Push Switch Rotary Encoder (482020514001, Wurth)
- 1 pc - Rotary Switch Knob (CL178862, Cliff)
- 1 pc - DIP Switch, 3 Way (NDS-03V, Diptronics)
- 1 pc - Fan, 25 x 25 mm, 12 V (MF25101V2-1000U-A99, Sunon)
- 1 pc - Fan, 80 x 80 mm, 12 V (MF80251V1-1000U-A99, Sunon)
- 1 pc - Sealed Ball Bearing, 8 x 22 x 7 mm (608ZZ, NSK)
- 1 pc - Sealed Ball Bearing, 12 x 28 x 8 mm (6001ZZ, NSK)
- 180 mm - Aluminum Profile, 20 x 20 mm (850-8476, RS PRO)
- 4 pcs - Round Rubber Foot, 21 x 4.2 mm (172-0867, RS PRO)
- 1 pc - El. Capacitor, 220 µF, 16 V (ECA1CM221, Panasonic)
- 1 pc - El. Capacitor, 1 µF, 63 V (860010772005, Wurth)
- 1 pc - Ce. Capacitor, 0.1 µF (C320C104K1R5TA, KEMET)
- 1 pc - N-Channel MOSFET, 10 A, 60 V (IRLZ14PBF, Vishay)
- 1 pc - Voltage Regulator, 1 A, 5 V (MC7805ACTG, Onsemi)
- 1 pc - Two-sided Prototyping Board (1160-EP, Rademacher)
- 1 pc - Full Size Breadboard (255-00006, RND Components)
- Add’l: Resistors, Cables, PCB Sockets, JST Connectors, etc.

Universal Fasteners:

- 1 pc - M12 Hex Nut & Washers [Right bearing]
- 1 pc - M8 x 15 mm Socket Screw & washers [Left bearing]
- 1 pc - M5 x 50 mm Socket Screw & nut [Rotation stop]
- 6 pcs - M5 x 15 mm Socket Screw [Heatsink & Alu. profile]
- 4 pcs - M5 x 10 mm Socket Screw [Sample holder brackets]
- 4 pcs - M4 x 30 mm Screw & Nut [Peltier fan]
- 2 pcs - M4 x 15 mm Socket Screw [Upper wheel bracket]
- 4 pcs - M3 x 25 mm Vinyl Screw & nut [PCB stack]
- 4 pcs - M3 x 20 mm Screw & Nut [Controller fan]
- 4 pcs - M2.5 x 6 mm Screw & Nut [Screen mount]
- 2 pcs - M2 x 8 mm Screw [Thermistor lugs]
- 3 pcs - No.6 x 1” Self-Tapping Screw [Right Leg]
- 2 pcs - No.6 x 5/8” Self-Tapping Screw [Right Leg]

3D Printed Parts:

Time and material estimates are calculated for a standard Creality Ender 3 S1 Pro running at 60 mm/s with 0.2 mm layer height, 0.4 mm nozzle diameter and support structures enabled. Use any standard 3D filament (PET-G, PLA, ABS, etc.) and normal perimeter/infill settings for all parts but Isolation.stl, for which high temperature filament (Polycarbonate) and minimal material settings gives higher insulation and a wider operational temperature range. The CAD files can be found at [5].

- 1 pc - RightLeg.stl [32 h, 340 g]
- 1 pc - FanShroud.stl [26 h, 200 g]
- 1 pc - LeftLeg.stl [11 h, 120 g]
- 1 pc - ControlPanel.stl [5 h, 45 g]
- 1 pc - BottomLid.stl [2 h, 25 g]
- 1 pc - UpperWheel.stl [2 h, 18 g]
- 1 pc - LowerWheel.stl [1 h, 8 g]
- 1 pc - TiltStop.stl [1 h, 10 g]
- 1 pc - PortCover.stl [10 m, 1 g]
- 1 pc - Isolation.stl [5 h, 50 g

Machined Metal Parts:

- 1 pc - SampleBasePlate.stp [Fig. S4]
- 2 pcs - SampleSideBracket.stp [Fig. S5]
- 1 pc - ThroughHoleSpindle.stp [Fig. S6]

Upload Compiled Binary File with Arduino IDE (V.1.8.19) in Windows 7 or newer

1. Connect the Arduino board to a computer via USB.
2. Start the Arduino IDE and open the Blink sketch: **File→Example→1.Basic**
3. Choose the correct board type and serial port: **Tools→Board/Port**
4. Turn on output at upload: **File→Preferences→Settings→Show Verbose Output During: Upload**
5. Upload the sketch: **Sketch→Upload**
6. In the IDE output panel, copy the AVR-Dude call, i.e. first line after the *Global variables use...* line.
7. For this command, replace the rightmost file path (*C:\Users\...\arduino build XXX/Blink.ino.hex:I*) with the absolute file path to the new binary file (*C:\Users\.../PeltierRockerNano.hex*).
8. For Windows 8 or newer: Put all three (3) file paths inside of quotation marks (-CC:\Users\.../avrdude.conf ⇒ -C”C:\Users\.../avrdude.conf”, etc.).
9. Open the Command Prompt (**Ctrl+Esc→”cmd”**), then paste and run the modified command.

CFD Simulation Details

The shear stresses were determined by solving the Navier-Stokes equations for laminar flow using a finite element software (*COMSOL Multiphysics 6.1*, COMSOL AB). To track and model the water-air interface, we used the phase-field method implemented in the software. Two simulation geometries were analyzed, a full 3D geometry of the rectangular well and a 2D planner symmetric geometry that corresponding to a vertical cut-plane along the centerline of the channel (culture plate well) and perpendicular to the rotation axis. At the solid walls, we used a no-slip boundary condition with contact angles of 70° and 0° along the walls and floor, respectively. The latter condition was chosen to reproduce the thin liquid film at the bottom that was observed in the experiments. To find a mesh that balances our computational capacity with our resolution requirements, a mesh convergence study was performed. This resulted in 3D and 2D meshes with maximum element sizes of 0.53 mm and 0.32 mm, as well as a total number of mesh elements of $1.5 \cdot 10^6$ and $5.3 \cdot 10^4$, respectively. To verify our simulation approach, we altered our 3D geometry to that of a circular culture plate and reproduced previous results [6] with an average shear stress of within 5%. When analyzing wavefront velocity, both the experiment and the simulations used water at 21°C. For the shear stress simulations water at 37°C was used to be more in line with a typical user application. The averaged shear stress presented in Fig. 4 was calculated after a consistent flow behavior was observed and for one full rocking period (four times the maximal rocking angle). The raw data in Fig. S10-S11 give full temporal insight into the wave propagation and resonance patterns. Finally, 3D simulations corresponding to the *Bt407* cells grown in 5 mL of water (growth conditions in Fig. 5B-C) were performed for both slow flow (4°/s) and fast flow (40°/s). These resulted in the shear stress distribution for the whole well, which can be seen time averaged over a full rocker period in Fig. S12.

Crystal violet staining protocol

We use a crystal violet staining protocol adapted from Stepanović et al. [7]. After biofilm incubation, the growth medium is carefully aspirated with a pipette. 3 mL of phosphate buffered saline (PBS) is added and carefully removed wash away remaining growth media. The biofilm is left for 20 min to allow it to dry, before adding 3 mL of 0.1% crystal violet solution for another min. The solution is then removed, and the wells washed with PBS 3 times, as described above. The wells are then filled with 3 ml 30% acetic acid for 20 min before collecting the biomass. We then measure the optical density (OD600) of the solution using a photometer (DEN-600, BioSan), diluting with deionised water as necessary for a final OD of <1.5. Photographs of biofilm are taken at the start of procedure as well as before adding the 30% acetic acid.

Statistical analysis

We performed statistical analysis on our data using Prism 10 (Prism 10.4, GraphPad Software). The 3 flow speeds (static, slow and fast) we compared for monolayer thickness and crystal violet assay results using one-way ANOVA. We have also made pair comparisons using Tukey's multiple comparisons. Stars on the graph indicate the degree of statistical significance of $p<0.05$ (*), $p<0.01$ (**) or non-significant $p \geq 0.05$ (ns).

Supplementary Materials

1. Goeres, D. M., Hamilton, M. A., Beck, N. A., Buckingham-Meyer, K., Hilyard, J. D., Loetterle, L. R., ... & Stewart, P. S. (2009). A method for growing a biofilm under low shear at the air–liquid interface using the drip flow biofilm reactor. *Nature protocols*, *4*(5), 783-788.
2. Ceri, H., Olson, M. E., Stremick, C., Read, R. R., Morck, D., & Buret, A. (1999). The Calgary Biofilm Device: new technology for rapid determination of antibiotic susceptibilities of bacterial biofilms. *Journal of clinical microbiology*, *37*(6), 1771-1776.
3. Williams, D. L., & Bloebaum, R. D. (2010). Observing the biofilm matrix of Staphylococcus epidermidis ATCC 35984 grown using the CDC biofilm reactor. *Microscopy and Microanalysis*, *16*(2), 143-152.
4. Schwartz, K., Stephenson, R., Hernandez, M., Jambang, N., & Boles, B. R. (2010). The use of drip flow and rotating disk reactors for Staphylococcus aureus biofilm analysis. *Journal of visualized experiments: JoVE*, (46), 2470.
5. Nilsson, D.P.G. PeltierRocker CAD. https://doi.org/10.6084/m9.figshare.25470136.v1 (2024)
6. Tucker, R. P., et al. "See-saw rocking: an in vitro model for mechanotransduction research" Journal of The Royal Society Interface 11.97 (2014): 20140330
7. Stepanović, S., Ćirković, I., Mijač, V., & Švabić-Vlahović, M. (2003). Influence of the incubation temperature, atmosphere and dynamic conditions on biofilm formation by Salmonella spp. Food Microbiology, 20(3), 339-343.
